# Infantile Subglottic Hemangioma: A Case Report

**DOI:** 10.31729/jnma.8500

**Published:** 2024-03-31

**Authors:** Richa Baniya, Ashlesha Chaudhary, Ashram Upadhyaya, Prashant Rijal

**Affiliations:** 1 Department of Paediatrics, Nepal Medical College and Teaching Hospital, Kathmandu, Nepal; 2 Nepal Medical College and Teaching Hospital, Kathmandu, Nepal

**Keywords:** *bronchoscopy*, *case reports*, *glottis*, *hemangioma*, *propranolol*

## Abstract

Subglottic hemangiomas are rare benign vascular tumors of infancy which involve the airway. It is a subtype of infantile hemangiomas and is a potentially life-threatening condition with a mortality rate of 50% if left untreated. Hence, early intervention in this condition is essential. Here we present a case of a 4-month-old infant, a male infant with a history of cough and noisy breathing requiring multiple hospital visits before eventually being diagnosed with subglottic hemangioma. Due to its similar presentation with other more common respiratory illnesses, the diagnosis can be missed. Oral propranolol is the first-line therapy, which was used successfully in our case.

## INTRODUCTION

Subglottic hemangioma, a type of infantile hemangioma is a rare condition with a prevalence of 1.76 cases per 100,000 live births, which are prone to get misdiagnosed.^1-3^ Hemangiomas involving the airway, particularly the subglottic region can be potentially life threatening because of airway obstruction. It is common for subglottic hemangioma to be initially misdiagnosed as croup especially in the absence of cutaneous hemangiomas and the treatment can be delayed similar to our case.^2^ Here, a four months old infant presenting with multiple episodes of respiratory symptoms is diagnosed as and treated for subglottic hemangioma.

## CASE REPORT

A 4-month-old male infant was referred to our centre with complaints of coughing, noisy breathing and fast breathing for 7 days. The patient had a similar history of cough and noisy breathing from the age of 2 months with symptomatic relief only during the duration of treatment. This was the patient's fourth episode that required the need for a hospital visit. In the previous hospital, the patient was diagnosed with bronchiolitis and managed as such. On examination, the infant was afebrile and had tachypnea with inspiratory stridor. Oxygen saturation was 94% in room air. Inspiratory stridor and conducted sounds were present on chest auscultation. Other systemic examination findings were normal.

The patient was admitted to the pediatric intensive care unit (PICU). All blood investigations were within normal limits. Ultrasonography of the neck revealed a nodular heterogeneously hyperechoic lesion adjacent to the left lobe of the thyroid. Contrast-enhanced computed tomography (CECT) was carried out which demonstrated a well-defined, heterogeneously enhancing soft tissue density at the subglottic region causing left posterolateral tracheal wall compression resulting in luminal narrowing with maintained airway patency. The thyroid gland was visualized separately from the lesion.

**Figure 1:**
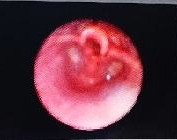
Bronchoscopy shows a soft, pinkish to purplish lesion causing a narrowing located in the subglottic region with maintained airway patency.

The thyroid function test and anti-thyroid peroxidase antibody test were normal. The patient was planned for bronchoscopy which revealed a soft, pinkish to purplish lesion causing a narrowing located in the subglottic region with maintained airway patency. The supraglottic structure, bilateral vocal cords, trachea and bronchial tree were normal. The bronchoscopy finding was suggestive of subglottic hemangioma (Figure 1).

The patient was started on oral propranolol 1 mg/kg/day and oral prednisolone at 1 mg/kg/day with continued PICU monitoring. After 3 days the patient's symptoms had started to reduce. CT Scan of abdomen showed no visceral hemangioma and echocardiography was normal. Patient was discharged on oral propranolol and oral steroid was gradually tapered and stopped. During follow-up after 6 months, the patient was playful with no complaints, and no respiratory symptoms. Magnetic Resonance Imaging (MRI) of the neck was done which was normal suggesting complete resolution of the subglottic hemangioma.

## DISCUSSION

Infants with subglottic hemangioma may present at birth and almost always present before 4 months of age. The diagnosis may be difficult owing to the rarity of the lesion and the similarity of presentation with other more common respiratory illnesses.^3^ Subglottic hemangiomas are associated with an alarming mortality rate that can reach 50% if left untreated and a high index of suspicion is needed.^4^ In our case, the patient was diagnosed with bronchiolitis and finally referred to our centre and further investigations showed a different condition. Hence, a high index of suspicion was crucial.

Imaging study is a less invasive method for diagnosing subglottic hemangioma. CT scans provide better visualization of the lesion and the degree of luminal narrowing.^5^ MRI with contrast can clearly show subglottic hemangioma and can differentiate the hemangioma from other vascular anomalies by demonstrating the internal architecture of the lesion.^6^ New modalities like three-dimensional (3D)- CT/bronchoscopy will reduce the need for invasive laryngoscopic studies and help to diagnose submucosal hemangiomas undetected on laryngoscope. In addition, 3D-CT/bronchoscopy will help to evaluate the extent of the lesion, degree of airway narrowing, and treatment response.^7^ With adequate sedation, bronchoscopy is ideal for the documentation of glottic and subglottic pathology.^8^

For a very long time, the management of infantile hemangiomas did not have adequate guidelines and consensus and the treatment was not standardized despite the presence of multiple case series and reviews.^9^ However, in 2019 the first clinical guideline for management for infantile hemangiomas^1^ was released which provided risk stratification and recognition of potentially problematic hemangiomas, early and frequent monitoring during the first few months of life to identify infantile hemangiomas that require intervention, role of imaging and evidence based guidance for management of infantile hemangiomas with both pharmacologic options and surgical modalities along with indications for consultation, referral and monitoring including parent education. Subglottic hemangioma has been categorized as life threatening hemangioma because it could obstruct the airway. Such hemangiomas are considered for early treatment and require further evaluation.^1^ Multiple medical and surgical interventions have been described thoroughly in many different literatures for the treatment of subglottic hemangiomas. Oral propranolol is the treatment of choice for infantile hemangioma including subglottic hemangioma.^1,9^ It is continued for at least six months and often maintained until 1 year of age or longer.^1^ Other intervention for subglottic hemangiomas include steroids, laser treatment, surgical excisions, tracheostomy or a combination of these therapies.^10^

## CONCLUSIONS

When an infant has signs or symptoms of coughing, wheezing, shortness of breath, difficulty in breathing, difficulty in feeding and so on, it is necessary for clinicians to have a broad list of differential diagnoses. Furthermore, if the same patient has multiple visits to the hospital with the same signs and symptoms, it is necessary to have a detailed investigation. Most infants with respiratory symptoms may have a common condition, however, sometimes a more ominous case of airway hemangiomas can be present. Therefore a thorough airway assessment is crucial.
